# A Quantitative Method for the Evaluation of Deep Vein Thrombosis in a Murine Model Using Three-Dimensional Ultrasound Imaging

**DOI:** 10.3390/biomedicines12010200

**Published:** 2024-01-16

**Authors:** Yanjun Xie, Yi Huang, Hugo C. S. Stevenson, Li Yin, Kaijie Zhang, Zain Husain Islam, William Aaron Marcum, Campbell Johnston, Nicholas Hoyt, Eric William Kent, Bowen Wang, John A. Hossack

**Affiliations:** 1 Department of Biomedical Engineering, University of Virginia, Charlottesville, VA 22908, USA; yx7pt@virginia.edu (Y.X.); yh5kd@virginia.edu (Y.H.); hcs5jsw@virginia.edu (H.C.S.S.); 2 Department of Surgery, School of Medicine, University of Virginia, Charlottesville, VA 22908, USA; kqj2aw@uvahealth.org (L.Y.); tgt8ek@uvahealth.org (K.Z.); zhi3tw@virginia.edu (Z.H.I.); wam3tk@virginia.edu (W.A.M.); zay7pw@virginia.edu (C.J.); neh5bx@virginia.edu (N.H.); eric.kent@emory.edu (E.W.K.); bw2pw@uvahealth.org (B.W.)

**Keywords:** 3D ultrasound, deep vein thrombosis, Doppler ultrasound, venous thromboembolism, pulmonary embolism

## Abstract

Deep vein thrombosis (DVT) is a life-threatening condition that can lead to its sequelae pulmonary embolism (PE) or post-thrombotic syndrome (PTS). Murine models of DVT are frequently used in early-stage disease research and to assess potential therapies. This creates the need for the reliable and easy quantification of blood clots. In this paper, we present a novel high-frequency 3D ultrasound approach for the quantitative evaluation of the volume of DVT in an in vitro model and an in vivo murine model. The proposed method involves the use of a high-resolution ultrasound acquisition system and semiautomatic segmentation of the clot. The measured 3D volume of blood clots was validated to be correlated with in vitro blood clot weights with an $R^{2}$ of 0.89. Additionally, the method was confirmed with an $R^{2}$ of 0.91 in the in vivo mouse model with a cylindrical volume from macroscopic measurement. We anticipate that the proposed method will be useful in pharmacological or therapeutic studies in murine models of DVT.

## 1. Introduction

Deep vein thrombosis (DVT) is a life-threatening condition that can lead to its sequelae pulmonary embolism (PE) and post-thrombotic syndrome (PTS), affecting over one million people in the United States annually [1]. The development of a thrombus in DVT obstructs blood flow; induces clinical manifestations such as edema, venous hypertension with pain, and ulceration; and has a detrimental effect on the quality of life of the patient [2]. DVT results in the use of extensive health resources and considerable social costs (USD 7 to 10 billion per year in the United States) [3].

Ultrasound is widely used in the detection of suspected DVT [4,5]. Doppler ultrasound or pulsed-wave ultrasound is used to determine the existence of blood clots when blood flow is occluded by blood clots and reduced to approximately zero [6,7,8]. However, in the field of rodent models of DVT, there is still a lack of a standard approach to the quantification of blood clots. Previous studies used the recanalization rate or blood flow restoration rate derived from two-dimensional (2D) Doppler/power Doppler ultrasound to qualitatively assess the efficacy of a treatment such as a recombinant tissue plasminogen activator (rtPA) [6,7]. The blood flow velocity in pulsed-wave ultrasound has been used to assess the influence of DVT [9,10]. However, it is highly dependent on the measured location and parameters such as the Doppler angle. Alternatively, the weight of the blood clots or the macroscopic observation of the length and width of the thrombus can be used as a quantitative measurement. This approach requires the harvest of clots from animals, which is a termination procedure [11,12]. Recent advances in high-resolution microscopic imaging have enabled the in vivo visualization of venous thrombus formation [13,14,15]. It is suitable for imaging the formation of DVT in a short time frame, since the vessels of mice must be exposed to light excitation. The invasive nature of surgery may restrict the use of this technique in research that involves multiple days of observation or in thrombolysis of subacute or chronic DVT.

Researchers frequently use ultrasound imaging-based approaches for the volumetric (i.e., full 3D) quantification of blood clots. A novel 3D imaging protocol was proposed to evaluate the volume echogenicity of the thrombus and was validated in clinical patients [16,17]. Additionally, a flow system was reported to assess in vitro thrombus volume during thrombolysis [18]. These studies obtained sequential 2D slices and generated estimates of the 3D volume of blood clots in clinical patients or in vitro models. Volumetric quantifications outperform 2D area-based methods, as they take into account the irregular shape of blood clots and do not require the imaging plane to be exactly aligned with the previous position [9,19]. Driven by these advantages, this work is the first application of 3D ultrasound examination of DVT assessment in murine models. It is distinct from previous research due to its requirement for a higher resolution than in clinical patients. Compared to existing in vitro models, imaging in in vivo models is challenging, as the surrounding tissues of the vessels may result in imaging artifacts or reduced contrast for DVT. This noninvasive ultrasound imaging technique can be used to further evaluate the most effective way to remove DVT by conducting thrombolysis studies in murine models.

In this paper, we present a 3D ultrasound approach for the quantitative assessment of a clot in a mouse model of DVT. To validate this quantification method, blood clots were first made and imaged using 3D ultrasound in an in vitro circulating system. The measured volume of blood clots was segmented and compared with their weight. The next step was to apply the approach in vivo in a mouse model of DVT and to make a comparison between the 3D volume of the blood clot from ultrasound and the macroscopic measurement at harvest. This work is significant in that it provides the first evidence that 3D ultrasound volume can be used as a nonterminating metric for assessing clot volume in a rodent DVT model.

## 2. Materials and Methods

### 2.1. In Vitro Blood Clot Model

**Figure 1:**
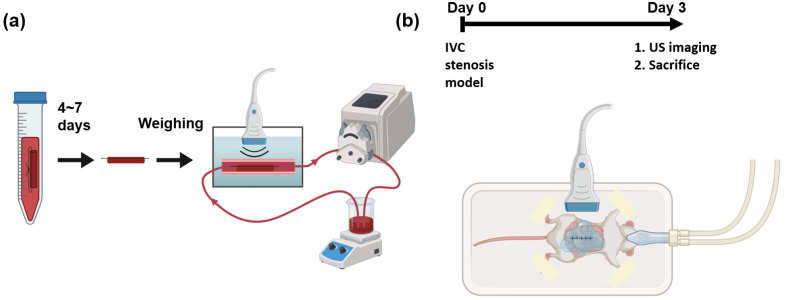
Schematic of experiments and imaging setups. (**a**) In vitro blood clots are fabricated and kept in refrigerator for 4 to 7 days. The weights of the blood clots are measured before running in a flow loop. The blood clots in whole bovine blood are imaged by a 3D ultrasound system. (**b**) A mouse of IVC stenosis model undergoes surgery at day 0. At day 3, the mouse is imaged using a 3D ultrasound system before sacrifice.

The experimental apparatus of the in vitro flow model is illustrated in Figure 1a. In vitro blood clots were formed with the addition of 15 mM calcium chloride to 3.8% citrated whole bovine blood (HemoStat Laboratories, Dixon, CA, USA) in 15 mL polypropylene conical tubes to reverse anticoagulation [20,21]. The mixture was placed into 2.5 mm diameter borosilicate glass tubes, and sutures were tied through the tubes. The mixture was incubated at 37 ${\;}^{\circ }$ C for 4 h. After 4 h, the samples were kept at 4 ${\;}^{\circ }$ C for 3 to 7 days to promote clot retraction [22,23]. Before experimentation, the clots were removed from the glass tubes but remained attached to the sutures. The weights of the clots were recorded after subtracting the weights of sutures of the same length that were inside the clots.

An in vitro experimental apparatus was employed to simulate the physiological environment of DVT. The apparatus was submerged in a 37 ${\;}^{\circ }$ C water bath. The blood clot with sutures was fixed in a segment of a thin-walled polyolefin tube (H&PC-58727, Sopoby, Wenzhou, China) of 3 mm inner diameter. Whole bovine blood (20 mL) was circulated within the flow loop using a peristaltic pump (NE-9000B, New Era Pump Systems, Inc., Farmingdale, NY, USA). The average velocity in the 3 mm inner diameter section was 47 mm/s, which is approximately equal to the blood flow velocity in the mouse vein [24]. An ultrasound transducer was submerged underwater in this apparatus.

### 2.2. Mouse Model of Deep Vein Thrombosis

All animal procedures conformed to the Guide for the Care and Use of Laboratory Animals [25] and study protocols approved by the Animal Care & Use Committee (ACUC) at the University of Virginia. C57BL/6 male mice (the Jackson Laboratory, Bar Harbor, ME, USA) approximately 8–10 weeks old were used in this study. The mouse vein underwent partial restriction of blood flow with minor modifications from previous articles [11,12,19]. A midline laparotomy was performed to dissect the inferior vena cava (IVC). Following permanent ligation of all side branches, a 5-0 proline suture string was placed on top of the IVC as a spacer, followed by ligation. The spacer was then removed, leading to a partial ligation of the IVC below the renal vein. The procedure resulted in a reduction of 90% in the lumen cross-section area at the ligation site [19]. The side branches were fully ligated, and the back branches were cauterized.

Three days after surgery, ultrasound imaging was performed to measure the volume of the blood clot. Ultrasound gel was applied to the abdominal region for coupling. After the imaging session, the clot was harvested for macroscopic measurement.

### 2.3. Ultrasound Imaging

Scanning 3D ultrasound volume can be accomplished by mechanically translating a 1D transducer and acquiring 2D frames at each location [26]. The Vevo 2100 system (Fujifilm VisualSonics, Inc., Bothel, WA, USA) was used with an external motor module (Fujifilm VisualSonics, Inc., Bothel, WA, USA) that mounted an MS-550D transducer to acquire 3D volumes of blood clots. During the imaging session, each mouse was fixed on a temperature-monitored heated motion stage (TM150, Indus Instruments, Webster, TX, USA) in the supine position while its nose was flushed under 1.0% *v*/*v* isoflurane (Henry Schein, Dublin, OH, USA). The transducer was operated at 40 MHz in B-mode and a focal zone of 6 to 8 mm with a 12 mm imaging depth. The location and orientation of the transducer were carefully adjusted to align the IVC within the imaging plane and provide the best image quality. During acquisition, the 3D volumetric data in B-mode and Doppler ultrasound were collected to segment blood clots from the background [4]. The step size was 0.03 mm along the long axis (sagittal plane) and 0.1 mm along the short axis (transverse plane), respectively. Ultrasound parameters are listed in Table 1. The schematic of the in vivo imaging setup is shown in Figure 1b.

### 2.4. Three-Dimensional Segmentation of Blood Clot

**Figure 2:**
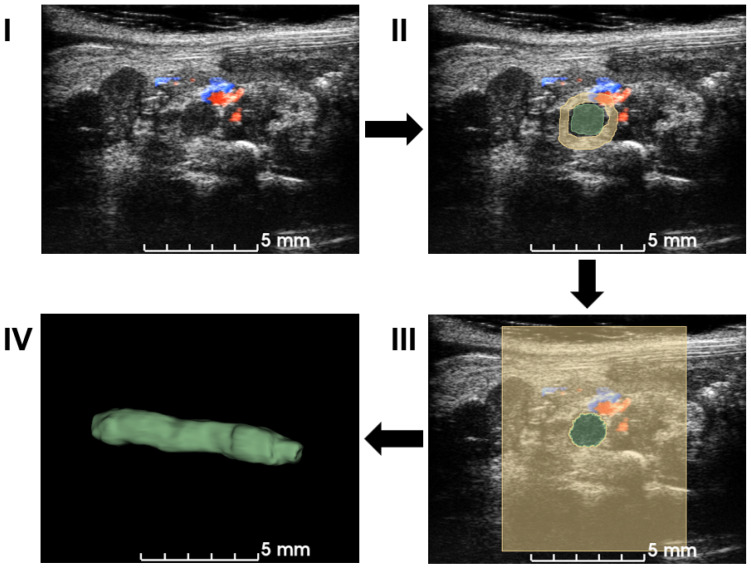
Workflow of the semiautomatic 3D segmentation. First, ultrasound volume is loaded into 3D slicer (**I**). Manual segmentation is performed on multiple slices and angles based on B-mode signal density and Doppler blood flow signal in (**II**). A seeding algorithm is run and the results are shown in a 2D view (**III**). Modifications are applied to extract a 3D volume of a blood clot in (**IV**). After the segmentation, the volume of the blood clot is calculated for quantification.

The workflow employed a semiautomatic GrowCut segmentation workflow [27], as illustrated in Figure 2. Volumetric data in the imaging system were exported to a PC and loaded into 3D slicer software (Version 5.0.3) for image analysis [28]. Blood clots and background in a few slices in each direction (X–Z, Y–Z, and X–Y planes) were manually annotated, which was supervised by an expert surgeon. The existence of the blood clot was based on the static texture and Doppler blood flow signals. After accumulating more than 30 annotations, a growing-from-seeds algorithm was run on a 3D slicer to segment the volume of the 3D blood clot [27]. After smoothing the boundary to the volume of 3D blood clots provided in the software, the volume of the blood clots was measured as a quantification metric.

As an alternative, a cylindrical volumetric estimation of the blood clot was used from macroscopic photos. The volume of the blood clot was calculated as follows [29]:

(1) $$V=\frac{\pi }{4}D^{2}L$$

where *D* and *L* are the diameter and length of the blood clot, respectively. The diameter in the macroscopic measurement included the thickness of the vein wall. To correct the blood clot diameter, vein wall thickness was estimated from ultrasound acquisition and subtracted from the macroscopic diameter.

### 2.5. Data Analysis

The statistical analysis was performed using MATLAB (2021a, Mathworks, Natick, MA, USA). The Pearson correlation coefficient $R^{2}$ was calculated using the inbuilt MATLAB corrcoef function [30].

## 3. Results

### 3.1. Three-Dimensional Blood Clot Volume and Weight

**Figure 3:**
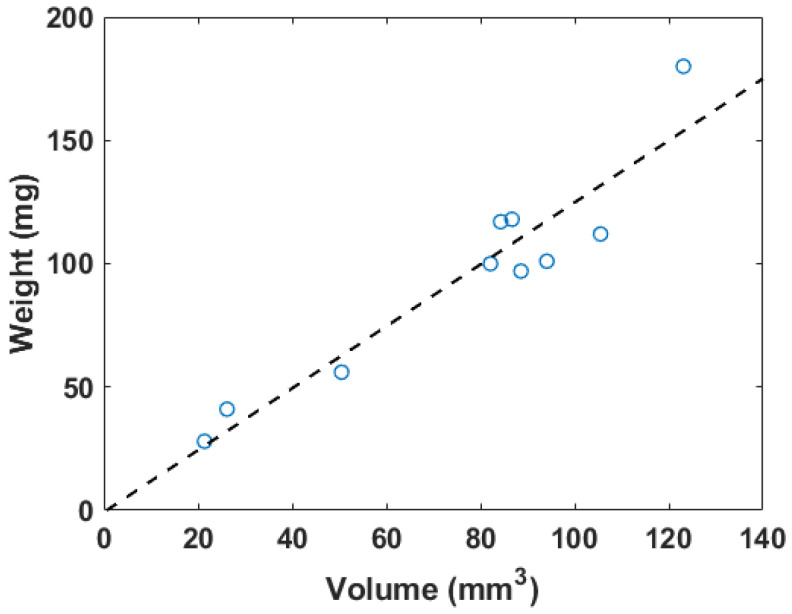
The correlation plot of in vitro blood clot weights versus 3D segmentation volume, where $R^{2}=0.89$ . The dashed line represents the linear regression result.

The weights measured before the flow loop and the total volumes are plotted in Figure 3. The inner diameter of the tubes holding the blood clots was 2.5 mm to ensure that the blood clots could be placed within the flow loop system. The weights of the blood clots ranged from 28 to 180 mg. The 3D volume measurement of the blood clot generated from the workflow described in Figure 2 varied from 21 to 123 ${\mathrm{mm}}^{3}$ . The dimension of blood clots was optimized for the field of view of the transducer and the limit of the 3D motion module.

The fitting linear relation between the weight (*W*) in mg and the 3D segmentation volume (*V*) in ${\mathrm{mm}}^{3}$ is described as

(2) W=1.2556V−0.7375.

**Figure 4:**
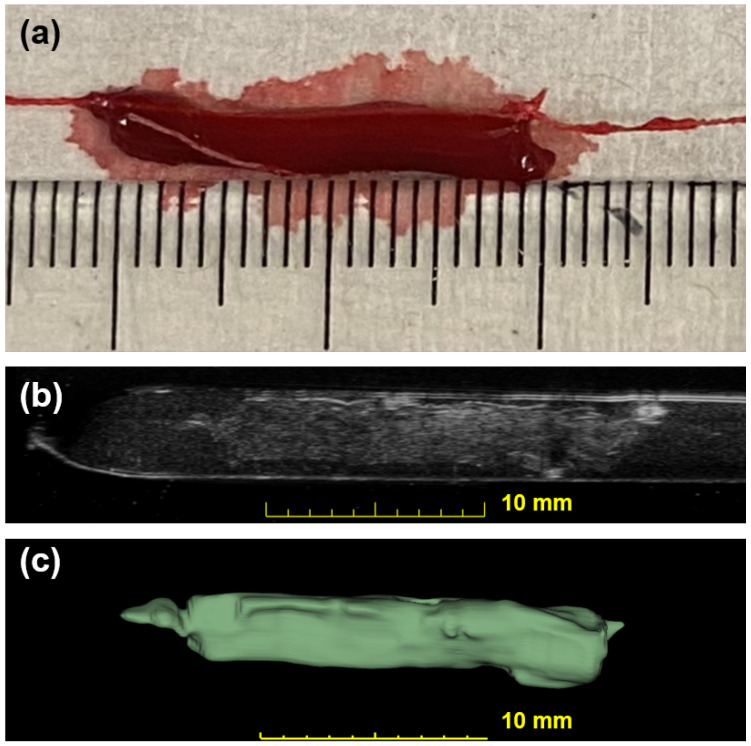
Photographic and ultrasonic examples of a bovine blood clot. (**a**) A photo of an in vitro blood clot. Each small tick on the ruler is equal to 1 mm. (**b**) One slice across the corresponding 3D US volume approximates the previous photo in (**a**). This clot has a weight of 101 mg and a volume of 94.1 ${\mathrm{mm}}^{3}$ . (**c**) The output segmentation volume of the clot is shown.

The $R^{2}$ for this linear fitting is 0.89. Figure 4 shows a photo of a blood clot with a mass of 101 mg and a volume of 94.1 ${\mathrm{mm}}^{3}$ . A cross-sectional slice was extracted in the 3D ultrasound volume after linear transforms. The texture and intensity difference distinguished the boundary of the blood clot from the background blood flow. The reconstruction of the 3D blood clot preserved correct geometry. Therefore, 3D ultrasound scanning is a suitable quantification readout to assess blood clot volume in nontransparent fluid media.

### 3.2. Characterization of Thrombus in Mouse of IVC Stenosis Model

**Figure 5:**
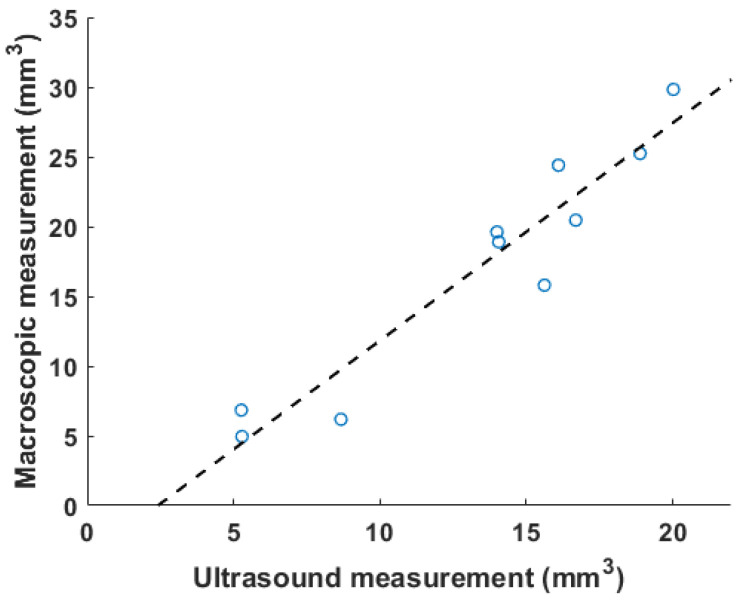
A correlation plot of in vivo results between volumetric measurement using ultrasound and macroscopic observation is plotted, with $R^{2}=0.91$ . The dashed line is the linear fitting result.

**Figure 6:**
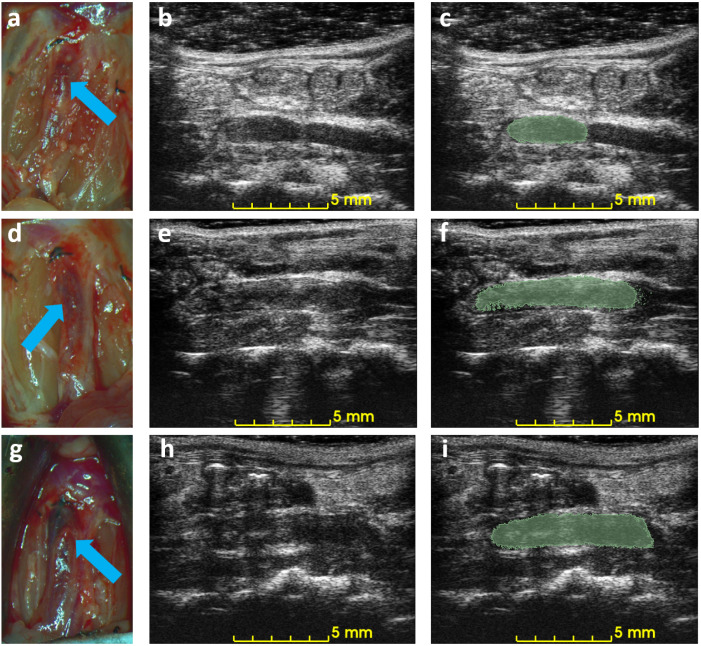
Example of macroscopic photos and ultrasound imaging on day 3. Each row is the macroscopic photo (**a**,**d**,**g**), the ultrasound image (**b**,**e**,**h**), and the ultrasound image with an overlaid mask of the blood clot (**c**,**f**,**i**) of each mouse. Arrows indicate the location of the detected blood clots in macroscopic photos.

Figure 5 shows the correlation between ultrasound volume and macroscopic observation. The volume of blood clots segmented from 3D ultrasound acquisition in mice ( N=10 ) ranged from 5.3 to 20.0 ${\mathrm{mm}}^{3}$ , and the mean volume was 13.5 ${\mathrm{mm}}^{3}$ . Meanwhile, the cylindrical volumes of the macroscopic measurements ranged from 5.0 to 29 ${\mathrm{mm}}^{3}$ after removing the mean thickness of the vein wall of 105 μ m from the macroscopic measurement. The correlation coefficient $R^{2}$ is 0.91. The root mean square error for this linear regression is 2.51 ${\mathrm{mm}}^{3}$ . Three examples of ultrasound imaging slices and their corresponding photos are shown in Figure 6. The thrombi are indicated with arrows in macroscopic observations and highlighted with light-green masks in ultrasound imaging, shown in Figure 6.

## 4. Discussion

In this study, a blood clot quantification method was developed to measure volume based on 3D B-mode and Doppler ultrasound imaging data. The 3D measurement method was applied to an in vitro flow loop model and a murine model with partial ligations. The volume of blood clots using a semiautomatic 3D segmentation algorithm was validated in the in vitro flow loop against their weight in volumes of 21.4 to 123 ${\mathrm{mm}}^{3}$ and weights of 28 to 180 mg, respectively. In the murine model, eight volumes of DVT from 5.3 to 20.0 ${\mathrm{mm}}^{3}$ were validated with macroscopic measurements.

Blood clots share similar density and incompressibility with water, which is one of the foundations for such a measurement. Vessels such as the IVC can be pressed and deformed. Most previous studies used color Doppler or pulsed-wave ultrasound to qualitatively determine the existence of blood clots [6,7]. To quantify changes before and after blood clot treatment, two-dimensional area-based measurements were also performed as an output metric [19]. The selection of 2D slice location is also subject to the operator’s expertise, and this benchmarking method does not take into consideration the irregular shape of the DVT outside of the imaging plane. Today, macroscopy is widely used to characterize a clot [11,12]. In our experiment, the volume of 3D ultrasound acquisition was compared with the macroscopic measurement. The cylindrical model of macroscopic measurement oversimplified the geometry of a blood clot [29]. The width of blood clots can vary from the center to the edges, and the shape of the cross section of blood clots in the short-axis view may not be circular. Finally, similar to the weighing method, it required the termination of mice. The above restrictions cause it to be an imprecise measure for quantifying blood clots, resulting in a larger volume than a genuine 3D technique. In Figure 5, the slope of the linear regression (1.4) is greater than 1. Unlike existing methods, the 3D volume method proposed in this paper was deformation-tolerated and was not subject to operations during acquisition. The abdominal area was completely recorded with a small step size, and a segmentation algorithm was run after acquisition. The high correlation coefficient (Figure 5) of 0.91 suggests that the 3D ultrasound volume method is a reliable measurement of DVT. Though all animals were treated with the same procedures, the blood clot volumes in Figure 5 are not evenly distributed within the range of 5 to 20 ${\mathrm{mm}}^{3}$ due to individual variability in the formation of the thrombus [12,19].

The 3D acquisition method can reduce the required number of animal subjects. Ultrasound is a noninvasive imaging technique that can be used to observe mouse IVC thrombus without causing any adverse effects, enabling the same subject to be monitored before and after treatment. The difference in volume is a reliable quantification of the efficacy of treatment in mice. A qualitative result or harvesting at each time point requires a larger number of mice in such an experiment. The use of 3D ultrasound in other medical conditions, such as plaque and abdominal aortic aneurysm, is already established [31,32,33]. However, 3D ultrasound is novel for assessing the rodent model of DVT, for which there is still no standard criterion for assessing DVT in a murine model. The proposed method has the potential to benefit DVT-related pharmacological or therapeutic in vivo studies by providing reliable quantified measurements throughout the treatment without any termination procedure. The difficulties of ultrasound imaging in the IVC thrombus are as follows: (1) obstructions from scarring, air, or food, for example, Figure 6h; (2) false color Doppler signals from the adjacent aorta; (3) absence of color Doppler signals due to slow blood flow. The challenges mentioned above, if not addressed, can affect the detection of the DVT boundary and lead to an inaccurate measurement, especially in traditional 2D B-mode ultrasound [34]. Acquisitions of 3D ultrasound provide additional data for thrombus evaluation, partially alleviating these concerns.

When the contrast of IVC ultrasound images was not sufficient, we employed some alternatives to ensure good imaging quality in mouse imaging, as follows: (1) Change the applied pressure of the transducer. The IVC was imaged at a different axial distance. (2) Change the imaging site. Shadowing of the ultrasound signal can be caused by air or food particles swallowed. We tilted the transducer angle away from the original plane where there were degrading imaging artifacts during data acquisition. These compensations mitigated the lack of signal contrast, thus reducing the chance of overestimation or underestimation of the volume of blood clots.

Occasionally, color Doppler imaging produces unreliable results, which can be caused by slow blood flow [34]. B-mode data were considered with color Doppler images simultaneously to avoid inaccurate segmentation. One sophisticated way to separate the static blood clot and tissue region from the dynamic blood flow is to apply a singular value decomposition (SVD) analysis to a multiframe data set at each position [35]. Therefore, it requires a longer acquisition time and a programmable system that can collect multiple frames at the same location between each mechanical translation. Another imaging technique that can be used is ultrasound elastography [36]. It can differentiate between blood clots and background due to the significant difference in stiffness. Recently, advances in deep learning have enabled automatic segmentation of DVT without annotations, which can be adopted and applied in the murine model in the future to simplify the procedure [37,38].

The seeding algorithm in Figure 2 needed a small number of manual annotations for the thrombus and background. It was mainly based on texture and intensity in the volumetric data. Segmentation of the blood clot did not guarantee a smooth boundary. After running the algorithm, we modified the mask to remove discrete clusters and cover the missing region. These modifications can generate a smoother 3D surface for blood clots.

The widely used Duplex ultrasound was proven to be a reliable and accurate technique to detect DVT [4,5]. To translate the proposed measurement method into clinical settings, an appropriate 3D ultrasound scheme is recommended. Existing 3D ultrasound measurements of DVT are based on the mechanical movement of the probe [16,17]. This mechanical movement can be achieved by either an internal or an external translation module or by performing a freehand scan. Motion correction techniques can be used in cases where there is substantial motion [39]. Advances in 2D matrix arrays have the potential to measure the volume of DVT in a single acquisition. However, 3D ultrasound imaging using a matrix array is usually associated with reduced resolution and demanding computational power for beamforming [40]. It is important to address these concerns before clinical applications.

## 5. Conclusions

We demonstrated a quantification method for DVT in an in vitro model and a murine model. The quantification method involved the use of a 3D ultrasound acquisition system and semiautomatic segmentation of a blood clot. The volume measured by ultrasound was validated with the weight of in vitro blood clots and the optical observation of an in vivo murine model of DVT. The correlation coefficients in the in vitro and in vivo models were 0.89 and 0.91, respectively, indicating a strong correlation. The technique does not require the euthanasia of animals and is noninvasive. The DVT volumes calculated using this method vary from 5 to 20 ${\mathrm{mm}}^{3}$ . We anticipate that 3D ultrasound measurement will be used to quantify pharmacological or therapeutic studies in murine models. The proposed method can accurately measure DVT and improve the data quality of future research.

## Figures and Tables

**Table 1:** Ultrasound imaging parameters in B-mode and Doppler mode.

Parameters	B-Mode	Doppler
Frequency	40 MHz	30 MHz
Depth	12 mm	
Width	14 mm	
Resolution (width × depth)	512 × 400 pixels	256 × 200 pixels
Step size	0.03 mm (long axis)/0.1 mm (short axis)	

## Data Availability

The data that support the findings of this study are available from the corresponding author upon request.
